# Stimulation-Induced Side Effects of Deep Brain Stimulation in the Ventralis Intermedius and Posterior Subthalamic Area for Essential Tremor

**DOI:** 10.3389/fneur.2021.678592

**Published:** 2021-06-09

**Authors:** Myung Ji Kim, Kyung Won Chang, So Hee Park, Won Seok Chang, Hyun Ho Jung, Jin Woo Chang

**Affiliations:** ^1^Department of Neurosurgery, Korea University Medical Center, Korea University College of Medicine, Ansan Hospital, Ansan-si, South Korea; ^2^Department of Neurosurgery, Brain Research Institute, Yonsei University College of Medicine, Seoul, South Korea

**Keywords:** deep brain stimulation, dysarthria, essential tremor, paresthesia, posterior subthalamic area, stimulation-induced side effect, ventralis intermedius

## Abstract

Deep brain stimulation (DBS) targeting the ventralis intermedius (VIM) nucleus of the thalamus and the posterior subthalamic area (PSA) has been shown to be an effective treatment for essential tremor (ET). The aim of this study was to compare the stimulation-induced side effects of DBS targeting the VIM and PSA using a single electrode. Patients with medication-refractory ET who underwent DBS electrode implantation between July 2011 and October 2020 using a surgical technique that simultaneously targets the VIM and PSA with a single electrode were enrolled in this study. A total of 93 patients with ET who had 115 implanted DBS electrodes (71 unilateral and 22 bilateral) were enrolled. The Clinical Rating Scale for Tremor (CRST) subscores improved from 20.0 preoperatively to 4.3 (78.5% reduction) at 6 months, 6.3 (68.5% reduction) at 1 year, and 6.5 (67.5% reduction) at 2 years postoperation. The best clinical effect was achieved in the PSA at significantly lower stimulation amplitudes. Gait disturbance and clumsiness in the leg was found in 13 patients (14.0%) upon stimulation of the PSA and in significantly few patients upon stimulation of the VIM (*p* = 0.0002). Fourteen patients (15.1%) experienced dysarthria when the VIM was stimulated; this number was significantly more than that with PSA stimulation (*p* = 0.0233). Transient paresthesia occurred in 13 patients (14.0%) after PSA stimulation and in six patients (6.5%) after VIM stimulation. Gait disturbance and dysarthria were significantly more prevalent in patients undergoing bilateral DBS than in those undergoing unilateral DBS (*p* = 0.00112 and *p* = 0.0011, respectively). Paresthesia resolved either after reducing the amplitude or switching to bipolar stimulation. However, to control gait disturbance and dysarthria, some loss of optimal tremor control was necessary at that particular electrode contact. In the present study, the most common stimulation-induced side effect associated with VIM DBS was dysarthria, while that associated with PSA DBS was gait disturbance. Significantly, more side effects were associated with bilateral DBS than with unilateral DBS. Therefore, changing active DBS contacts to simultaneous targeting of the VIM and PSA may be especially helpful for ameliorating stimulation-induced side effects.

## Introduction

Deep brain stimulation (DBS) is a safe and effective treatment for medically refractory essential tremor (ET) (1). The nucleus ventralis intermedius (VIM) of the thalamus has been used as a primary target for DBS (2). However, proximal postural tremors and distal intention tremors are often refractory to VIM DBS. Several studies exploring potential targets for DBS have reported promising results for the posterior subthalamic area (PSA) with respect to tremor suppression (3–10), particularly for tremors that are difficult to control with VIM DBS (5, 6). The PSA, including the zona incerta, prelemniscal radiation, and cerebellothalamic tract (containing the dentatorubrothalamic tract) (11, 12), has been suggested as a potentially effective target for DBS to treat ET. With the advancement of surgical techniques, targeting the PSA by advancing the electrode deeper along the appropriate trajectory from the VIM is now possible (5, 13–15). Since the VIM and PSA are located at different contacts along the same electrode, this approach allows for a comparison of the two targets in terms of tremor reduction and stimulation-induced side effects. The investigation of stimulation-induced side effects is necessary, particularly with respect to the PSA, as the destruction of the PSA by lesioning has been associated with significant adverse events (16–18). Therefore, in the present study, we analyzed and compared stimulation-induced side effects and tremor reduction associated with DBS targeting the VIM and PSA *via* a single electrode based on individual active contacts.

## Materials and Methods

### Patients

In the present study, patients with medically refractory ET who were implanted with a single DBS electrode simultaneously targeting the VIM and PSA at our hospital between July 2011 and October 2020 were retrospectively reviewed. Patients who were followed up for <6 months and those diagnosed with tremors other than ET (such as dystonia tremor or multiple sclerosis tremor) were excluded. This study received ethical approval from the institutional review board of our institution.

### Surgical Procedure

The surgical technique used in this study has been described previously (13). The operation involved frame-based stereotactic implantation of a DBS electrode that simultaneously targeted the VIM and PSA. Stereotactic 1.5 T magnetic resonance imaging (MRI) was performed preoperatively, and the data were transferred to the Leksell SurgiPlan (Elekta, Stockholm, Sweden). Standard stereotactic coordinates for VIM localization were as follows: 13–15 mm lateral to the midline and 25–28.5% of the length of the anterior commissure-posterior commissure line anterior to the posterior commissure in the intercommissural plane. PSA localization was verified using MRI and the Schaltenbrand atlas. After localizing the targets, the angle of the trajectory necessary to advance the electrode to the PSA between the subthalamic nucleus and the red nucleus was determined using T2-weighted MRI. Trajectory planning was performed using the VIM as the primary target. The coronal and sagittal angles were adjusted as needed to create a trajectory that hit the PSA target, and procedures to evaluate the effect of stimulation on tremors and possible side effects were performed under local anesthesia. During surgery, the ventral thalamic border was identified using microelectrode recordings. The electrode was then advanced to a location that was 5–6 mm below the ventral thalamic border, and a test stimulation was initiated to evaluate tremor reduction and identify any side effects. Previously, microelectrode recordings have been used to indirectly locate the PSA based on the verification of the motor-evoked firing of VIM neurons and tremor cells inside the VIM nucleus of the thalamus. For permanent stimulation, DBS electrodes (model 3387, Medtronic, Minneapolis, MN, USA) were used. Based on the microelectrode recording results, contacts 0 and 1 were located in the PSA, and contacts 2 and 3 were located in the VIM. After the electrodes were implanted, postoperative computed tomography (CT) was performed before the frame was removed, and the scans were merged with preoperative MR images to determine the positions of the electrodes. Lastly, an implantable pulse generator (Soletra, Activa SC, Activa PC, or Activa RC, Medtronic) was implanted subcutaneously in the infraclavicular region under general anesthesia during the same session.

### DBS Contacts and Parameters

**Figure 1:**
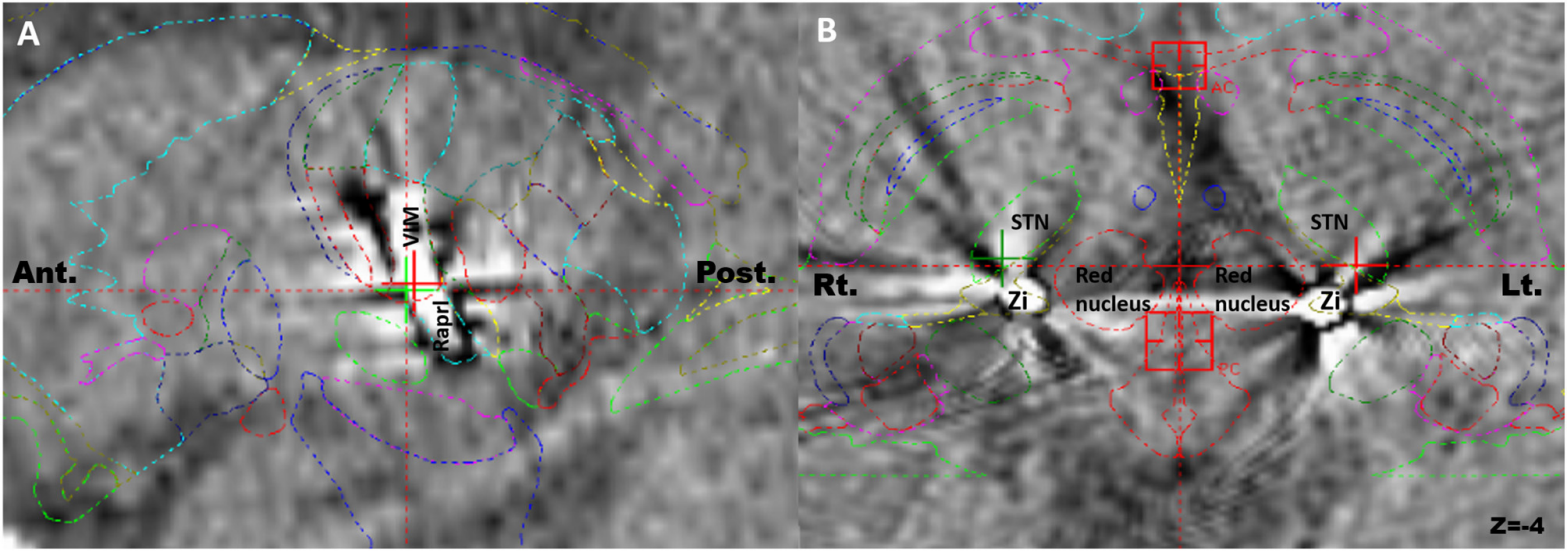
Postoperative computed tomography scans merged with preoperative magnetic resonance images for identification of the actual electrodes and active contacts using Schaltenbrand atlas. **(A)** The electrode hit the ventralis intermedius and the prelemniscal radiation simultaneously in the sagittal plane. **(B)** The tips of bilateral electrodes were located in the zona incerta in the axial plane. Ant, anterior; Post, posterior; Rt, right; Lt, left.

During the first programming session, contact 0 or 1 (PSA) was activated, followed by contact 2 or 3 (VIM) for either single or double monopolar stimulation. In the case of bilateral electrodes, two electrodes were simultaneously activated in the same way. The effect of stimulation on each contact was evaluated to determine stimulation-induced side effects. Lastly, dual activation of contact 0 or 1 and contact 2 or 3 (VIM + PSA) was performed in all patients. The active contacts with the best clinical effects (tremor reduction in the contralateral hand) and the fewest side effects were analyzed. The contacts displaying the best effect were chosen for chronic stimulation. The effect of each electrode on the tremors in the contralateral hand was evaluated separately. The amplitude, frequency, and pulse width were modulated using the optimal therapeutic window to improve the tremor. More complex stimulation paradigms, such as interleave or bipolar settings, were chosen if needed. Based on the stimulation parameters required for tremor suppression in each patient, group comparisons were performed. Importantly, even if a surgeon plans a trajectory that hits the PSA and VIM, the final location of the electrode could be altered due to surgical errors, intraoperative adjustments according to micro- or macrostimulation, and/or trajectory modifications to avoid vessels. The final location of the active contacts that were chosen for chronic stimulation were verified using the postoperative CT scan merged with the preoperative MRI image and the Schaltenbrand atlas using the Leksell SurgiPlan (Figure 1). For further analysis, stereotactic surgical planning was performed using Stealth Station S8 (Medtronic) according to previous target coordinates for each electrode. The planning data were uploaded into SureTune 3 (Medtronic) and merged with the postoperative CT data. Anatomical structures (e.g., VIM, zona incerta, subthalamic nucleus, substantia nigra pars reticulata, and red nucleus) were identified to reveal the relationship between stimulation-induced side effects and the location of the electrodes.

### Tremor Outcomes

Patients were evaluated according to the Clinical Rating Scale for Tremor (CRST) preoperatively and at 6 months, 1, and 2 years after DBS electrode implantation. As 22 patients received bilateral DBS, the effect of each electrode was evaluated separately. The CRST subscores for the treated upper extremity were calculated by adding the scores of all single items pertaining to that extremity from parts A and B of the CRST (19). The “writing” item was only included for the dominant hand, leading to maximum possible scores of 32 or 28 points per extremity. This evaluation using the CRST was performed according to the methodology presented by Stacy et al. (20).

### Statistical Analysis

Changes in CRST subscores were evaluated using a linear mixed model in the MIXED procedure of SAS (version 9.4, SAS INC., Cary, NC, USA). The analysis used repeated measures data obtained from each patient with no input for missing data because the follow-up period varied for each patient. To determine whether a statistical difference in CRST scores existed between the groups over time, the interaction between group and time was evaluated. In addition, to visualize changes in CRST scores over time, least-square means and standard errors for each time point were obtained to show the mean profile plot. The analysis of variance and Kruskal-Wallis tests were used to compare variables. The Fisher's exact test was used to compare the stimulation-induced side effects of each contact. All *p*-values were two-tailed, and statistical significance was set at *p* < 0.05.

## Results

### Baseline Characteristics

A total of 97 patients underwent DBS for ET control between July 2011 and October 2020. Four patients with follow-up periods of <6 months, two patients who did not undergo follow-up clinical evaluation after DBS, and one patient who was diagnosed with multiple sclerosis were excluded from this study. Finally, the present study included 93 patients with 115 implanted DBS electrodes (71 unilateral and 22 bilateral). Patient demographics are shown in Table 1. With regard to stimulation parameters, the median amplitude, pulse width, and frequency were 2.4 V, 80 μs, and 160 Hz, respectively.

**Table 1:** Patient demographics and stimulation parameters.

Age^*^	62.9 ± 7.8
Follow-up duration^**^ (months)	38 [16,65]
Sex^***^	
Male	76 (78.4%)
Female	21 (21.6%)
Uni/Bilateral^***^	
Unilateral	71 (73.2%)
Bilateral	22 (22.7%)
Baseline CRST subscore	19.0
Amplitude^**^	2.4 [1.9, 2.8]
Pulse width^**^	80 [60, 90]
Frequency^**^	160 [130, 160]

* *Values are presented as mean ± SD*.

** *Values are presented as median [Q1, Q3]*.

*** *Values are presented as percentage*.

*CRST, Clinical Rating Scale for Tremor*.

### Location of Active Contacts for Chronic Stimulation

Among the 115 electrodes, 210 active contacts were identified; in 37.8, 29.4, 25.9, and 6.9% of the patients, the contacts 1, 0, 2, and 3 were chosen for chronic stimulation, respectively (Table 2). Based on the lead analysis, the most stimulated structure was the zona incerta (43.8%), followed by the VIM (27.6%) and the prelemniscal radiation (24.2%). Notably, our analysis revealed that the surgical procedure had good accuracy in terms of positioning the intended targets to hit the VIM and PSA simultaneously.

**Table 2:** Locations of active contacts for chronic stimulation.

Electrode	115
PSA (contact 0 or 1)	55 (47.8%)
VIM (contact 2 or 3)	9 (7.8%)
PSA + VIM (contact 0 or 1 and contact 2 or 3)	51 (44.4%)
Active contacts	210
0	62 (29.4%)
1	79 (37.8%)
2	54 (25.9%)
3	15 (6.9%)
**Location**	
Zi	92 (43.8%)
VIM	58 (27.6%)
Raprl	51 (24.2%)
Vop	6 (2.9%)
STN	2 (1.0%)
Voa	1 (0.5%)

*PSA, posterior subthalamic area; Raprl, preleminiscal radiation; STN, subthalamic nucleus; VIM, ventral intermediate nucleus of the thalamus; Voa, ventrooralis anterior nucleus of the thalamus; Vop, ventrooralis posterior nucleus of the thalamus; Zi, zona incerta*.

### Tremor Reduction and Stimulation Parameters for Chronic Stimulation

**Figure 2:**
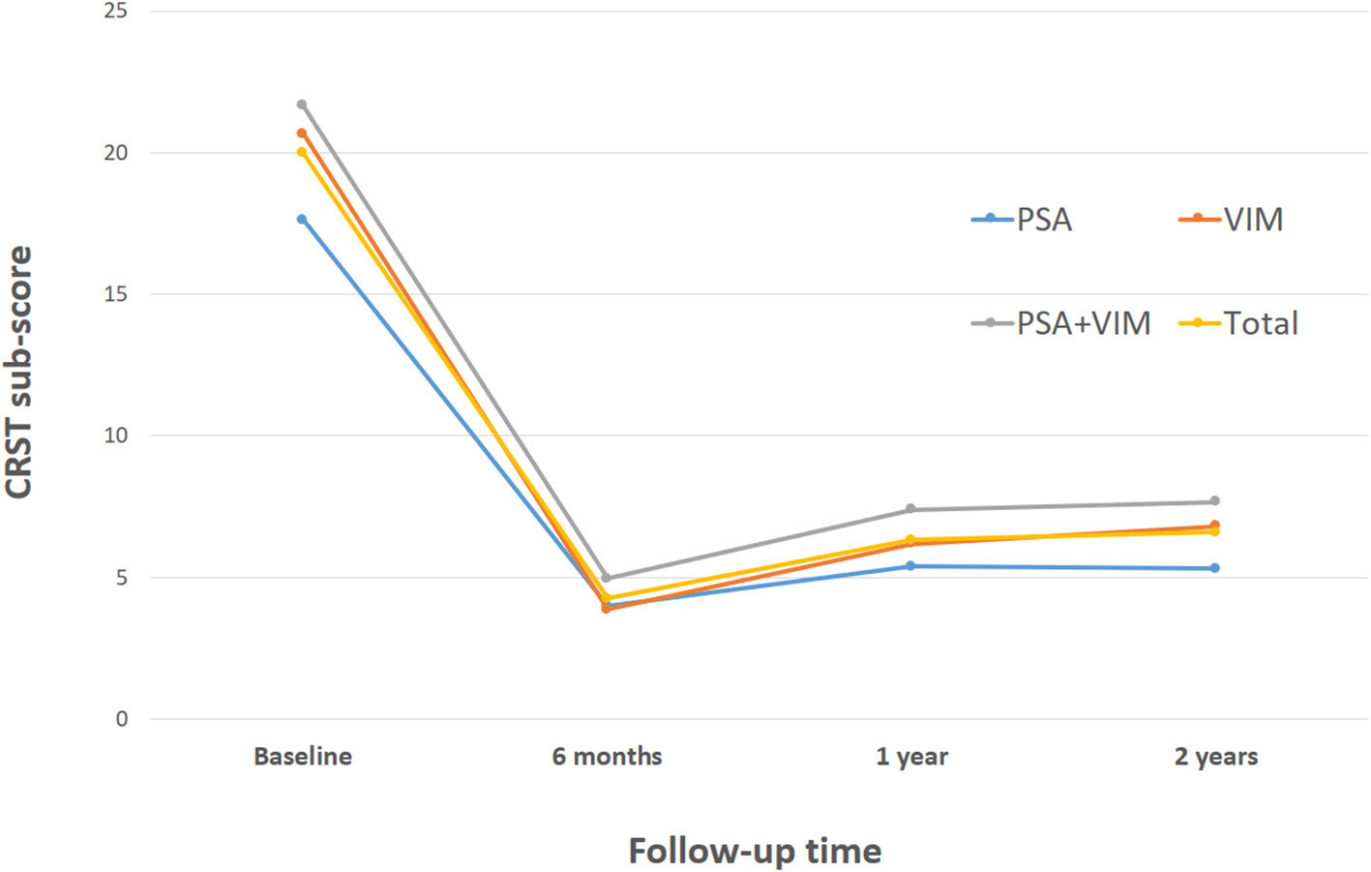
Changes of Clinical Rating Scale for Tremor (CRST) subscores over time. CRST subscores at evaluation visits. The graphs represent LSmeans and SE of the CRST subscores at different evaluation visits. PSA, posterior subthalamic area; VIM, ventral intermediate nucleus of the thalamus.

The overall CRST subscore decreased from 20.0 at baseline (*N* = 115) to 4.3 (78.5% decrease), 6.3 (68.5% decrease), and 6.5 (67.5% decrease) at the 6 month (*N* = 115), 1 year (*N* = 93), and 2 year (*N* = 65) follow-ups, respectively (Table 3). A significant difference was observed in CRST subscores over time (*p* < 0.001). The least-square means of the CRST subscores were significantly different among the groups at baseline, with a score of 17.6 in the PSA, 20.7 in the VIM, and 21.7 in the VIM + PSA (Table 3). The CRST subscore decreased from 17.6 to 4.0 (77.3% decrease) in the PSA, from 20.7 to 3.9 (81.2% decrease) in the VIM, and from 21.7 to 5.0 (77.0% decrease) in the VIM + PSA at the 6 month follow-up. However, the CRST subscore increased slightly after 6 months in all three groups. Although the CRST subscores among the three groups were not statistically significant over time, chronic stimulation 2 years after DBS of the PSA (5.3, 70.0% decrease from baseline) resulted in slightly better tremor control than that after DBS of the VIM (6.8, 67.1% decrease from baseline) and the VIM + PSA (7.7, 64.5% decrease from baseline). Figure 2 shows the mean profile plot of the changes in CRST subscores over time among the three groups. The mean amplitude, pulse width, and frequency for chronic stimulation were 2.1 V, 79.7 μs, and 149.6 Hz, respectively, in the PSA; 3.1 V, 81.7 μs, and 153.3 Hz, respectively, in the VIM; and 2.7 V, 87.6 μs, and 161.3 Hz, respectively, in the VIM + PSA (Table 4). The best clinical effect was achieved with the PSA at significantly lower stimulation amplitudes and frequencies (*p* = 0.002 and *p* = 0.016, respectively).

**Table 3:** Clinical Rating Scale for Tremor (CRST) subscores over evaluation visits.

**Follow-up**	**Total (*****N*** **= 115)**		***p*-value (time)**	**PSA (contact 0 or 1)**		**VIM (contact 2 or 3)**		**PSA + VIM (contact 0 or 1 and 2 or 3)**		***p*-value (group and time)**
	**CRST estimate**	**SE**		**CRST estimate**	**SE**	**CRST estimate**	**SE**	**CRST estimate**	**SE**	
Baseline	19.9815	0.6426	–	17.6306	0.8913	20.7267	2.1853	21.6665	0.9577	**0.0095**
6 months	4.2570	0.4380	**<0.001**	4.0150	0.6342	3.8712	1.4714	4.9715	0.7353	0.5914
1 year	6.3256	0.5629	**<0.001**	5.3927	0.7832	6.1902	1.7618	7.4118	0.9446	0.2662
2 years	6.5970	0.5799	**<0.001**	5.3210	0.8351	6.8074	1.5768	7.6784	1.0359	0.3105

*CRST, Clinical Rating Scale for Tremor; PSA, posterior subthalamic area; SE, standard error; VIM, ventral intermediate nucleus of the thalamus. Boldface type indicated statistical significance (p < 0.05). The change in CRST subscores was evaluated using a linear mixed model*.

**Table 4:** Chronic stimulation parameters.

**Parameters**	**PSA (*N* = 55)**	**VIM (*N* = 9)**	**PSA+VIM (*N* = 51)**	***p*-value**
	**Mean ± SD**	**Mean ± SD**	**Mean ± SD**	
Voltage	2.1 ± 0.8	3.1 ± 1.8	2.7 ± 0.7	**0.024**
Pulse width	79.7 ± 20.1	81.7 ± 23.2	87.6 ± 28.8	0.391
Frequency	149.6 ± 16.2	153.3 ± 19.7	161.3 ± 16.9	**0.016**

*PSA, posterior subthalamic area; VIM, ventral intermediate nucleus of the thalamus. Boldface type indicated statistical significance (p < 0.05)*.

### Stimulation-Induced Side Effects

**Figure 3:**
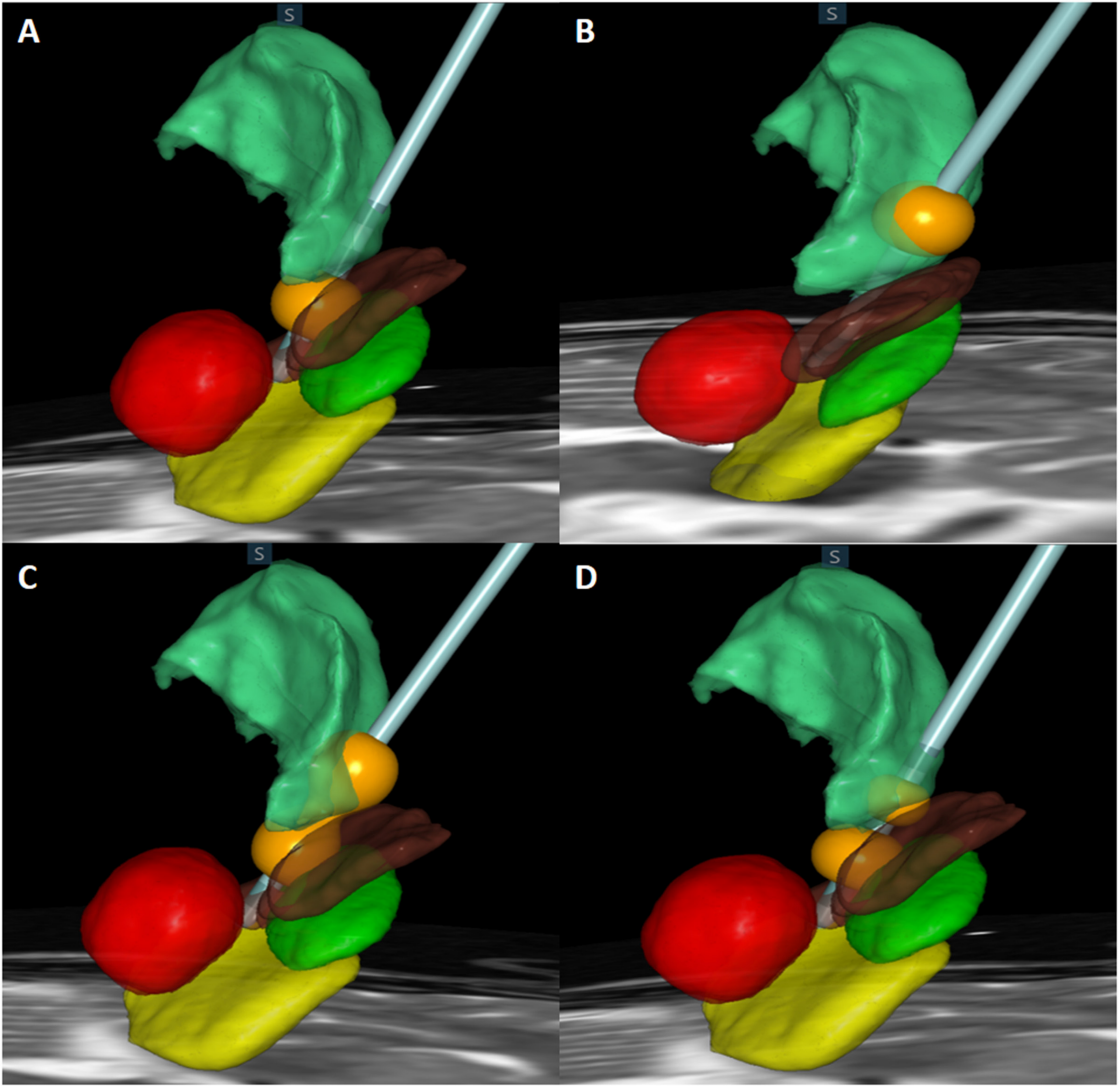
Actual electrode and contact of stimulation in relation to the subthalamic nucleus (STN) (green), substantia nigra (SNr) (yellow), red nucleus (red), zona incerta (Zi) (brown), and ventralis intermedius (VIM) (light green) are shown. **(A)** Monopolar stimulation of contact 1 located in the Zi. **(B)** Monopolar stimulation of contact 3 located in the VIM. **(C)** Dual stimulation of contact 1 in the Zi and contact 3 in the VIM. **(D)** Bipolar stimulation of contacts 1 and 2.

Table 5 shows the stimulation-induced side effects of each contact in the 93 included patients. A total of 13 patients (14.0%) reported gait disturbance and reduced leg control when either contact 0 or 1 below the intercommissural line (ICL) was stimulated (Figure 3A); this number was significantly higher than that when contact 2 or 3 above the ICL was stimulated (*p* = 0.0002). Dysarthria occurred in 14 patients (15.1%) when contact 2 or 3 was stimulated (Figure 3B); this number was significantly higher than that when contact 0 or 1 was stimulated (*p* = 0.0233). Transient paresthesia occurred in 13 patients (14.0%) after stimulation below the ICL and in six cases (6.5%) after stimulation above the ICL. Gait disturbance and dysarthria occurred significantly more frequently in those undergoing bilateral DBS than in those undergoing unilateral DBS (31.8% vs. 8.5%, *p* = 0.00112 and 45.5% vs. 11.3%, *p* = 0.0011, respectively). Paresthesia resolved either after a reduction in amplitude or change to bipolar stimulation without any loss of optimal tremor control. To reduce the side effect of gait disturbance due to stimulation below the ICL and dysarthria due to stimulation above the ICL, some sacrifice of optimal tremor control was required at that particular electrode contact. These side effects were reversible when changing the active contact to dual VIM + PSA stimulation (Figure 3C) or to bipolar stimulation (Figure 3D).

**Table 5:** Stimulation-induced side effects on each contact.

**Stimulation-induced side effect**	**PSA (contact 0 or 1) *N* (%)**	**VIM (contact 2 or 3) *N* (%)**	***p*-value**	**Unilateral DBS (total 71) *N* (%)**	**Bilateral DBS (total 22) *N* (%)**	***p*-value**
Gait disturbance	13 (14.0%)	0 (0%)	**0.0002**	6 (8.5%)	7 (31.8%)	**0.0112**
Dysarthria	4 (4.3%)	14 (15.1%)	**0.0233**	8 (11.3%)	10 (45.5%)	**0.0011**
Paresthesia	13 (14.0%)	6 (6.5%)	0.1448	15 (21.1%)	4 (18.2%)	1.00

*Boldface type indicated statistical significance (p < 0.05)*.

*DBS, deep brain stimulation; ICL, intercommissural line; PSA, posterior subthalamic area; VIM, ventral intermediate nucleus of the thalamus*.

## Discussion

In the present study, we investigated tremor outcomes and stimulation-induced side effects of DBS targeting the VIM and PSA *via* a single electrode. The strengths of our study are the large number of patients (115 implanted DBS electrodes in 93 patients with ET) and evaluation of long-term outcomes. In the present study, dysarthria, gait disturbance, and paresthesia were the most common stimulation-induced side effects, consistent with previous reports (21–23). We identified that single electrode DBS targeting both the PSA and VIM can be used when stimulation-induced side effects occur.

### Clinical Outcomes

PSA (contact 0 or 1) was most often chosen for chronic stimulation followed by VIM + PSA and VIM (Table 2). Tremor improved from baseline at all time points (Table 3). Additionally, favorable outcomes in terms of overall improvement in CRST subscores for the treated side were observed in this study, and among the three groups, there was no significant difference in tremor suppression with respect to each patient's individual active contacts (Table 3). The VIM required a significantly lower stimulation amplitude (Table 4). Additionally, although tremor was less effectively controlled over time with VIM + PSA stimulation (Table 3), the least-square means of CRST subscores were significantly higher at baseline with VIM + PSA stimulation. These findings suggest that the VIM and PSA should be chosen as the active contacts for patients with severe tremors.

### Dysarthria

The most common stimulation-induced side effect associated with VIM DBS was dysarthria (Table 5). A meta-analysis reported that the most commonly reported speech disorder following thalamic DBS was dysarthria (24.2%) (24). Dysarthria has been frequently observed after VIM DBS (18, 25, 26), most likely due to its effects on the corticobulbar fibers of the internal capsule (27–31). The unintended lateral spread of current can also activate the corticospinal tract and subsequently lead to involuntary muscle contraction of the arms and/or legs as well as lead to dysarthria (32, 33). With VIM DBS, this is often considered a consequence of excessively lateral electrode placement, affecting the internal capsule (18, 34). Notably, in the present study, stimulation-induced dysarthria was ameliorated by changing active DBS contacts to either VIM + PSA or bipolar stimulation.

### Paresthesia

Gait disturbance and paresthesia were more commonly associated with stimulation of the PSA (Table 5), and paresthesia was usually transient. However, when paresthesia persisted, it could be eliminated by adjusting DBS parameters, such as a reduction in amplitude or bipolar stimulation. Paresthesia is the most common side effect of stimulation of the medial lemniscus, posterior in the subthalamic area (31, 33), and the spread of electric current to the ventral caudal thalamic nucleus, which is posterior to the VIM (25, 30, 32). Paresthesia exacerbated by the spread of electric current away from the VIM can be ameliorated by a more anterior placement of the electrode within the VIM (35, 36). Sensory side effects are often considered susceptible to habituation over time and less prone to impede the treatment results (37). Paresthesia can be overcome with programming adjustments (38). Paresthesia can be diminished by decreasing the amplitude of stimulation since it is voltage dependent. Our findings suggest that a slow, gradual increase in amplitude and the use of bipolar stimulation to minimize the spread of current to the nearby medial lemniscus are effective in ameliorating stimulation-induced paresthesia.

### Gait Disturbance

In 10 patients, stimulation *via* active contacts in the PSA (0 and 1) was changed to VIM + PSA stimulation due to stimulation-induced gait disturbance and reduced leg control despite the loss of optimal tremor control. Previous studies have also observed side effects of PSA stimulation, which mainly included stimulation-induced gait ataxia and clumsiness of the contralateral lower limb (18, 39). Stimulation of the cerebellothalamic tract has also been shown to cause postural instability and gait ataxia. These symptoms can be attributed to chronic VIM/PSA stimulation leading to maladaptive plasticity of different fiber tracts (vestibulocerebellar-thalamic afferents and cerebello-rubrospinal tracts) (26, 40–42). More posterior and medial stimulation could activate the cerebellothalamic tracts, leading to gait disturbance or ataxia (16, 32). Cerebellar symptoms, including hypotonia, dysmetria, and gait disturbance or imbalance, were often reported after ablation of the subthalamic dorsal area (18, 43–45). Although the destruction of the PSA by lesioning has been reported to be associated with significant adverse events (43, 45), in the present study, no severe adverse events were observed during the evaluation of PSA DBS. This may be because while PSA DBS overrides tremor oscillations, it does not interrupt patterns of information related to proprioceptive sensations (46). Previous studies have suggested that large pulse width stimulation might account for DBS-induced cerebellar side effects and have recommended short pulse width settings for DBS (47–49). Another important issue is that patients with ET often have concomitant cerebellar ataxia, a phenomenon recently classified as ET plus syndrome (32, 50). Baseline ataxia may become more apparent after a successful reduction in tremor through DBS.

### Unilateral vs. Bilateral

Gait disturbance and dysarthria were significantly more frequent in those undergoing bilateral DBS than in those undergoing unilateral DBS (Table 5). Previous studies have also reported that stimulation-induced side effects are more frequently observed after bilateral procedures than after unilateral procedures (18, 51–55), with a 2- to 3-fold higher risk of dysarthria and ataxia associated with bilateral procedures (24, 32, 36, 52, 56). However, bilateral stimulation is more effective than unilateral stimulation for treating severe bilateral tremors and tremors combined with midline axial tremors (e.g., head tremors) and voice tremors (57). When treating axial tremors that require bilateral DBS, careful evaluation of long-term benefits and risks that may affect the patient's quality of life is essential, and staged operations should be considered at times. Since we activated bilateral electrodes simultaneously for severe bilateral tremors and axial tremors in this study, it was not possible to determine the effect of each electrode individually. Furthermore, it was difficult to determine each electrode's effect on dysarthria and gait disturbance. Therefore, since numerous stimulation-induced side effects associated with bilateral stimulation have been reported, our center recently changed our protocol so that the electrode on the contralateral side with respect to severe tremors is activated first and the other electrode is activated in a delayed manner.

### Limitations

The current study has several limitations. A major limitation is that it was a retrospective review of a single institution's clinical practice. Randomized controlled trials comparing the VIM and PSA directly through “on-off” stimulation of each contact are necessary to confirm our conclusions. Second, the follow-up period varied for each patient. The CRST score was evaluated preoperatively (*N* = 115 electrodes) and again at 6 months (*N* = 115), 1 year (*N* = 93), and 2 years (*N* = 65) postoperation. To compensate for this weakness, we adopted a linear mixed model. Further investigations with continuous follow-up are necessary to confirm the long-term effects of VIM and PSA DBS on tremor reduction, as well as to assess tolerance. Third, only parts A and B of the CRST, which were objective measurements rated by an experienced examiner, were evaluated. Part C of the CRST, which includes a patient-reported measurement of functional disability due to the tremor, and the Essential Tremor Questionnaire, assessing quality of life in relation to the tremor, were not included since these were subjective measurements. Further studies are needed to determine functional disability and quality of life in patients with ET undergoing DBS. Lastly, while we identified that more adverse side effects were associated with bilateral stimulation than with unilateral stimulation, we failed to confirm the effect of either electrode individually or according to target as both electrodes were activated simultaneously. Therefore, prospectively designed studies are needed to confirm our conclusions.

## Conclusion

In the present study, we analyzed tremor outcomes and stimulation-induced side effects in a large sample of patients (115 DBS electrodes in 93 patients with ET) who underwent DBS targeting both the VIM and PSA using a single electrode. Favorable results in terms of overall tremor improvement, stimulation-induced side effects, and surgical accuracy for the intended targets were observed. Knowing the different stimulation-induced side effects associated with the PSA and VIM and their effects on the results of the treatment is essential. The most common stimulation-induced side effects associated with VIM DBS and PSA DBS were dysarthria and gait disturbance, respectively. These side effects were significantly more common in those undergoing bilateral DBS than in those undergoing unilateral DBS. Additionally, in this study, we found that changing active DBS contacts to simultaneous targeting of the VIM + PSA may be especially helpful for ameliorating stimulation-induced side effects.

## Data Availability Statement

The raw data supporting the conclusions of this article will be made available by the authors, without undue reservation.

## Ethics Statement

The studies involving human participants were reviewed and approved by Severance Hospital Clinical Trial Institutional Review Board. Written informed consent for participation was not required for this study in accordance with the national legislation and the institutional requirements.

## Author Contributions

JC: conceptualization, writing (review and editing), and resources. MK: visualization and roles/writing (original draft). KC: data curation and formal analysis. SP: methodology and software. WC: project administration investigation. HJ: supervision and validation. All authors contributed to the article and approved the submitted version.

## Conflict of Interest

The authors declare that the research was conducted in the absence of any commercial or financial relationships that could be construed as a potential conflict of interest.
